# Acknowledgment of Reviewers 2024

**DOI:** 10.1089/heq.2024.80057.revack

**Published:** 2025-02-21

Critical evaluations of articles by expert reviewers make a vital contribution to ensuring the high quality of a journal’s content. The editorial leadership of *Health Equity* is very grateful for the support of the highly qualified peer reviewers who have dedicated their time and effort to reviewing our articles. We would like to show our appreciation by thanking the following individuals for their assistance with the review of articles for the journal in 2024.*

*Data as of November 22, 2024.

Sarah Abboud

Emily Adam

Robyn B. Adams

Ellesse Akre

Sirry Alang

Jenifer Allsworth

Amy Alspaugh

Carmen Alvarez

Michele Andrasik

Shervin Assari

Megan Attridge

Nadvadee Aungkawattanapong

Jesus M. Barajas

Bridget Basile Ibrahim

Jay Behel

Leia Belt

Darlene Bhavnani

Christopher Blackwell

Luisa Blanco

Bridgette E. Blebu

Ulrike Boehmer

Emily Boniface

Natalie Bradford

Angela Branch-Vital

Henna Budhwani

Miranda Buhler

Jake Bush

Vanessa Byams

Melanie Canterberry

Cristian J. Chandler

Zhuo Chen

Carolyn Clancy

Kate Clancy

Caroline Compretta

Eileen Condon

Monika Damle

Elisheva Danan

Jamie Daw

Amber DeJohn

Sheila Desai

Jane Desborough

Clara Dismuke-Greer

Andrew C. Dixon

Margaret Mary Downey

Cornelius Dyke

Kristen Eckstrand

Erica Eliason

Kerstin Emerson

Brian J. Fairman

Moshtagh R. Farokhi

Cristina Fernandez

Karen Florez

Justin Fontenot

Liza Fuentes

Taleria Fuller

Coral Gartner

Marybeth Gasman

Cory Gatrall

Mary Gerend

Alex K. Gertner

Stephanie Gilbertson-White

Sarah Gollust

Neera Goyal

Janessa Graves

Claire Greene

Kenneth Gruber

Amy Hagopian

Jing Hao

Margaret Hargreaves

Idethia S. Harvey

Megan Henly

Leah Hoffman

Louisa M. Holmes

Xiaolong Hou

Grace Howard

Xin Hu

Yen-Ming Huang

Marie-Fatima Hyacinthe

Maia Ingram

Ans Irfan

Elizabeth Jarpe-Ratner

Jessica Jensen

Tesheia Johnson

William M Jones

Michael R. Kauth

Reena Kelly

Sophie Kemper

Ladawan Khaikham

Manorama Khare

Heeun Kim

Christian King

Leah Koenig

Sira Korpaisarn

Kevin Kovach

Tonette Krousel-Wood

Tony Kuo

Kristen Landrum

Abigail Lara

Raeann Leblanc

Sharon Leitch

Emily Lemon

Edwin Lindo

Chen Liu

Lacy Loomer

Julie E. Lucero

Teralynn Ludwick

Yona Lunsky

Huabin Luo

Audrey Lyndon

Wei Lyu

Lisa N. Mansfield

Marjan Mardani-Hamooleh

Cassondra Marshall

Airín Martinez

Aresha Martinez-Cardoso

Steven Martino

Luz Adriana Matiz

Amanda C. McClain

Mollie McDermott

Ted McDonald

Martin McKee

Catherine Meads

Luis. D. D. Medina

Dara Mendez

Aaloke Mody

Diana Montoya-Williams

Vanesa Mora Ringle

Ethan Morgan

Megan Morris

Heidi Moseson

Amanda Nagle

Sunny Nakae

May Navarra

Monika Nayak

Chemen M. Neal

Gila Neta

Kristian Niemi

Tabashir Nobari

Ejiro Ntekume

Serwaa Omowale

Jan Oosting

Kene Orakwue

Robin L. Page

Evelina Pappa

Jamie Parnes

Amy Paterson

Yinan Peng

Jacob Perlson

April Pettit

Arrianna Planey

Julian Ponce

Jenny Potter

Laura Axelrod Potter

Neeraj Puro

Maya Ragavan

Aditi Ramakrishnan

Schenita Randolph

John J. Reynolds-Wright

Nat Roberts

Will Ross

Deborah Jane Russell

Anne Sadler

Gerardo Santos-Lopez

Diksha Sapkota

Teresa Schmidt

Rebecca Schwei

Emma Seagle

Salma Shariff-Marco

Mona Shattell

Madeleine Shea

Lu Shi

Deirdre Shires

Fitriana Kurniasari Solikhah

Chao Song

Jewel Stafford

Carl Streed

Mitch Stripling

Osama Tanous

Victoria L. Tiase

Elisabet Tiselius

Nathaniel Tran

Mary-Jo Trepka

Anna Valdez

Luis Valdez

Brandon Varilek

Alisa Velonis

Timothea Vo

Shannon Walker

Junling Wang

Ruoxi Wang

Xutong Wang

Ena Wanliss

Darren L. Whitfield

Emily Williams

Michelle Wong

Brad Wright

Heather Wurtz

Xin Yin

Matthew Yu

Lu Zhang

Wenhui Zhang

Yuejen Zhao

